# Nomograms predict prognosis and hospitalization time using non-contrast CT and CT perfusion in patients with ischemic stroke

**DOI:** 10.3389/fnins.2022.912287

**Published:** 2022-07-22

**Authors:** He Sui, Jiaojiao Wu, Qing Zhou, Lin Liu, Zhongwen Lv, Xintan Zhang, Haibo Yang, Yi Shen, Shu Liao, Feng Shi, Zhanhao Mo

**Affiliations:** ^1^Department of Radiology, China-Japan Union Hospital of Jilin University, Changchun, China; ^2^Department of Research and Development, Shanghai United Imaging Intelligence Co., Ltd., Shanghai, China

**Keywords:** CT, perfusion, prognosis, ischemic stroke, nomograms

## Abstract

**Background:**

Stroke is a major disease with high morbidity and mortality worldwide. Currently, there is no quantitative method to evaluate the short-term prognosis and length of hospitalization of patients.

**Purpose:**

We aimed to develop nomograms as prognosis predictors based on imaging characteristics from non-contrast computed tomography (NCCT) and CT perfusion (CTP) and clinical characteristics for predicting activity of daily living (ADL) and hospitalization time of patients with ischemic stroke.

**Materials and methods:**

A total of 476 patients were enrolled in the study and divided into the training set (*n* = 381) and testing set (*n* = 95). Each of them owned NCCT and CTP images. We propose to extract imaging features representing as the Alberta stroke program early CT score (ASPECTS) values from NCCT, ischemic lesion volumes from CBF, and TMAX maps from CTP. Based on imaging features and clinical characteristics, we addressed two main issues: (1) predicting prognosis according to the Barthel index (BI)–binary logistic regression analysis was employed for feature selection, and the resulting nomogram was assessed in terms of discrimination capability, calibration, and clinical utility and (2) predicting the hospitalization time of patients–the Cox proportional hazard model was used for this purpose. After feature selection, another specific nomogram was established with calibration curves and time-dependent ROC curves for evaluation.

**Results:**

In the task of predicting binary prognosis outcome, a nomogram was constructed with the area under the curve (AUC) value of 0.883 (95% CI: 0.781–0.985), the accuracy of 0.853, and F1-scores of 0.909 in the testing set. We further tried to predict discharge BI into four classes. Similar performance was achieved as an AUC of 0.890 in the testing set. In the task of predicting hospitalization time, the Cox proportional hazard model was used. The concordance index of the model was 0.700 (SE = 0.019), and AUCs for predicting discharge at a specific week were higher than 0.80, which demonstrated the superior performance of the model.

**Conclusion:**

The novel non-invasive NCCT- and CTP-based nomograms could predict short-term ADL and hospitalization time of patients with ischemic stroke, thus allowing a personalized clinical outcome prediction and showing great potential in improving clinical efficiency.

**Summary:**

Combining NCCT- and CTP-based nomograms could accurately predict short-term outcomes of patients with ischemic stroke, including whose discharge BI and the length of hospital stay.

**Key Results:**

Using a large dataset of 1,310 patients, we show a novel nomogram with a good performance in predicting discharge BI class of patients (AUCs > 0.850). The second nomogram owns an excellent ability to predict the length of hospital stay (AUCs > 0.800).

## Introduction

Stroke is classically characterized by neurological deficits resulting from acute focal vascular-related damage to the central nervous system (Sacco et al., 2013). Data from the Global Burden of Diseases (GBD) suggested that, in 2019, stroke remained the second-leading cause of death and the third-leading cause of death and disability combined, with the majority (80%) of cases attributed to an ischemic etiology (Go et al., 2014). Over the last decade, along with deeper insights into the disease, effective interventions for the major risk factors and the application of more effective treatments have led to a significant decline in stroke-related mortality. However, there are still a number of patients with stroke suffering from long-term disability, which increases the financial burden on both individual and societal levels (Di Carlo et al., 1999).

NCCT, non-contrast CT; CTP, CT perfusion; ADL, activity of daily living; GBD, Global Burden of Diseases; BI, Barthel index; ASPECTS, Alberta stroke program early CT score; CBF, cerebral blood flow; CBV, cerebral blood volume; MTT, mean transit time; TTP, time to peak; LASSO, least absolute shrinkage and selection operator; BSR, best subset regression; AIC, Akaike information criterion; CNN, convolutional neural network; ANN, artificial neural networks; XGB, extreme gradient boosting; GBM, gradient boosting machine; ROC, receiver operating characteristics; AUC, area under the curve; C-index, concordance index; OR, odds ratio; HR, hazard ratio; CI, confidence interval.

The activities of daily living (ADLs) have been considered one of the most basic indicators to evaluate the functional status of patients with stroke, which is also the main treatment objective for rehabilitation (Quinn et al., 2011). The Barthel index (BI) is one of the most widely used and most widely studied ADL evaluation methods, and it can not only be used to assess the functional status before and after treatment but can also be used to predict the outcomes of treatment, length of hospital stay, and prognosis (Mahoney and Barthel, 1965; Roberts and Counsell, 1998; Duncan et al., 2000; Sangha et al., 2005; Quinn et al., 2009; Li et al., 2020).

Non-contrast computed tomography (NCCT) scan is typically the first neuroimaging test performed in patients with suspected stroke and has the advantages of rapid detection, low cost, and few contraindications (Wardlaw et al., 2004; Timpone et al., 2020). However, acute tissue changes are difficult to be detected by NCCT in cases where ischemic symptoms resolve within 24 h, with a detection rate of only 4% (Douglas et al., 2003; Foerster et al., 2012). Therefore, other neuroimaging techniques such as brain CT perfusion (CTP) are always indicated after the NCCT imaging given its insensitivity. With the growing interest in the significance of cerebral physiology changes over time in clinical decision-making for ischemic stroke, CTP is always employed as a rapid and practical examination technique to identify and differentiate potentially salvable risk tissues (“penumbra”) and irreversibly damaged tissues (infarct “core”) through a relative quantitative measure of brain perfusion, thereby not only enhancing the understanding of cerebral physiology processes of stroke but also providing guidance on stroke management strategies (e.g., endovascular procedures) (Krishnan et al., 2017). Despite challenges in the standardization and accuracy of quantitative assessment, CTP is evolving as a cornerstone for imaging-based strategies in the rapid management of ischemic stroke (Mendelson and Prabhakaran, 2021).

In the last decade, numerous advances have been made in the field of feature selection methods, which can capture features from target images, thereby providing more radiological details to assist in clinical decision-making (Mayerhoefer et al., 2020). Currently, feature selection methods like radiomics technology have been applied in the field of stroke after the evaluation and selection of independent features (Chen et al., 2021; Wang et al., 2021), whose major applications include infarction detection (Peter et al., 2017), thrombosis characterization (Hasan et al., 2019; Qiu et al., 2019; Hofmeister et al., 2020), identification of high-risk carotid plaque (Zhang et al., 2021), and prediction of malignant middle cerebral artery infarction (Wen et al., 2020). The above examinations are all based on NCCT or CT angiography (CTA), whereas CTP is still mainly employed for the diagnosis of stroke by providing a relatively accurate estimation of the volume of infarction and ischemic penumbra. Recently, machine learning has been jointly employed in clinical practice to solve critical problems regarding outcome prediction in ischemic stroke (Feng et al., 2018). Certain cases employ magnetic resonance imaging (MRI) consisting of two modalities: MR perfusion similar to CTP and diffusion weighted imaging (DWI), whereas other studies adopted CT data for clinical prediction of ischemic stroke (Ho et al., 2017, 2019; Pinto et al., 2018). However, there has been no relevant research on the application of CTP-based radiomics technology regarding prognosis prediction in patients with stroke yet.

Due to the relatively complex course of a stroke, prognosis prediction of patients with stroke has always been a hot topic that draws a great number of interests. Thus, we conducted this retrospective study to determine whether nomograms based on imaging and clinical features can predict ADL and length of hospital stay in patients with ischemic stroke, with the aim of providing appropriate therapeutic and management strategies.

## Materials and methods

### Patients

**FIGURE 1:**
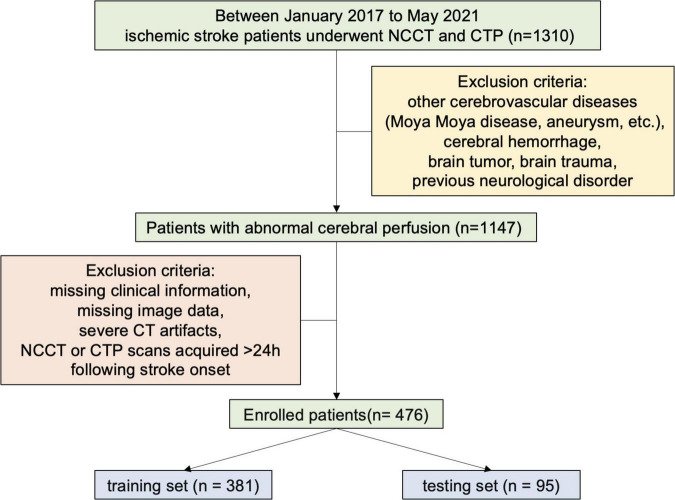
Patient sample inclusion flowchart. NCCT, non-contrast CT; CTP, CT perfusion.

The current diagnostic study received approval from the Institutional Ethics Committee of our hospital. The study was performed in accordance with the 1964 Declaration of Helsinki and its later amendments. From January 2017 to May 2021, a cohort of 1,310 patients diagnosed with ischemic stroke in our institution were included in this study. The participants had abnormal cerebral perfusion and could live independently before the infarction. NCCT and CTP scans were acquired within 24 h following stroke onset. Cases with other cerebrovascular diseases (Moyamoya disease, aneurysm, etc.), cerebral hemorrhage, brain tumor, brain trauma, previous neurological disorder, missing clinical or image data, and severe CT artifacts were excluded. Finally, a total of 476 patients were selected and divided into the training set (*n* = 381) and the testing set (*n* = 95) by the stratified sampling method, in which the discharge BI distribution of the training set was the same as that of the testing set (Figure 1).

All patients underwent a whole-brain stroke CT protocol using SOMATOM Force scanner (Siemens Healthineers AG, Erlangen, Germany) and Revolution scanner [General Electric Company (GE), Chicago, CA, United States], including thin-slice NCCT (≤2 mm) and CTP. During follow-up, endovascular thrombolysis or mechanical thrombectomy was performed to treat the ischemic stroke.

### Clinical characteristics

The clinical information of samples was collected, including the age, sex, BI of admission and discharge, risk factors for cerebrovascular disease (dyslipidemia, stroke history, cerebral infarction, atrial fibrillation, hyper-homocysteinemia, diabetes, hypertension, and smoking), brain injury, complications (hemorrhagic infarction, brain edema, epilepsy, dysphagia, and deep venous thrombus), therapeutic modality, and time for hospitalization. Importantly, the primary outcome in this study was a good functional outcome, defined as the BI higher than 60 at the date of discharge representing the good performance in ADL. The BI could be divided into four classes, in which scores of 0–40 indicated “total dependency,” 41–60 indicated “severe dependency,” 61–99 indicated “moderate dependency,” and 100 indicated “independent.”

### Imaging characteristics

All images were processed using a research portal platform.^1^ (1) NCCT images analysis: For patients with ischemic stroke, the Alberta stroke program early CT score (ASPECTS) could be used to evaluate the early changes in middle cerebral artery territory (MCAT) (Cao et al., 2022). Briefly, the brain was segmented into 20 ASPECTS regions. An embedded deep learning algorithm provides a 0 or 1 score as to whether an infarct occurs in each region. As features, we obtained ASPECTSs for the left brain, the right brain, and the whole brain, respectively, ranging from 0 to 10. Meanwhile, the mean CT intensity (unit: HU) was also collected for all regions. Note that the ASPECTS values were evaluated automatically based on our previous proposed deep learning algorithm (Cao et al., 2022). (2) CTP images analysis: The raw CTP images were calculated, and parameter maps were obtained. For features, we collected the volumes in these parameter maps including relative cerebral blood volume (CBV) (using threshold of <34, <38, and <42%), relative cerebral blood flow (CBF) (using threshold of <20, <30, <34, and <38%), time to top (*T*_max_; using threshold of >4, >6, >8, and >10 s), mismatch, and mismatch ratio.

### Binary clinical outcome prediction (discharge BI > 60 vs. BI ≤ 60)

First, univariate and multivariate logistic regression analyses were performed to evaluate the independent important factors for promoting a good outcome (discharge BI > 60) in the training set. Considering multicollinearity among the imaging variables, two variables were removed to get the best model. Independent clinical factors and imaging characteristics from the multivariate logistic regression model were integrated into the nomogram for predicting the presence of a good outcome.

The performance of the constructed nomogram was assessed in terms of three aspects: discrimination capability, calibration, and clinical utility. Receiver operating characteristics (ROC) curves and confusion matrixes were plotted, and the area under the curve (AUC) was calculated quantitatively. Then, calibration curves were also used to confirm the discrimination capability and accuracy of the proposed nomogram in both the training and testing sets. Moreover, decision curve analyses and clinical impact curves were conducted at different threshold probabilities for the training and testing sets, showing clinical net benefit for predicting outcomes.

### Multivariate clinical outcome prediction (four classes)

Four-class (BI: 0–40, 41–60, 61–99, and 100) classification task was conducted. First, each feature underwent *z*-score normalization. Then, feature selection was performed in the training dataset. During the procedure of feature selection, two operators were used orderly, as *F*-test’s *p*-value of 0.05 and the least absolute shrinkage and selection operator (LASSO) with α of 0.05. The output feature’s number was 47 and 9, respectively. With the sequential execution of two algorithms, the optimal features avoiding collinearity and overfitting could be finally obtained. After the feature selection, the BOX-COX transformation and the machine learning classifier (Bagging decision tree) were applied to construct the model. The predicted model was also tested and characterized by ROC curves and confusion matrixes.

### Hospitalization time prediction using Cox proportional hazard model

In order to establish the Cox proportional hazard model, we should ensure that the status was a good outcome (discharge BI > 60), and the time was the duration in the hospital, meaning that once the patient achieved a good outcome, he/she could be discharged. To select the optimal features, three methods were applied. The first one was the COX model, in which univariate and multivariate Cox regression analyses were performed to evaluate the independent important factors for promoting the good outcome (discharge BI > 60) in the training set. The second one was the best subset regression (BSR) model. Considering of the maximum adjusted *R*^2^ and the minimum Akaike information criterion (AIC), the best feature subset was selected to classify the four classes. The third one was the LASSO model to reduce the dimensionality of input features. Based on the three feature subsets, three Cox proportional hazard models were established, named COX, BSR, and LASSO, respectively.

When comparing the performances of the three models, LASSO was finally chosen in terms of the higher concordance index (C-index) and fewer features. Then, the nomogram was based on the proportional conversion of each regression coefficient from the multivariate Cox regression to a 0- to 100-point scale, which could be used to predict the time of hospitalization. Calibration curves and time-dependent ROC curves for the nomogram were calculated in the training and testing sets.

### Statistical analysis

All variables were summarized by using descriptive statistics, in which the counts and percentages were calculated for categorical variables and median, 25% quantile, and 75% quantile were used for continuous variables. Significant differences between the training set and testing set were performed by the Mann–Whitney *U*-test for continuous variables and the chi-square test for categorical variables, and a *p*-value < 0.05 was considered statistically significant. Univariate and multivariate binary logistic regression analyses were used to identify important factors related to the discharge BI. Univariate and multivariate Cox regression analyses were used to determine features related to the time of patients to achieve a good outcome. In univariate analyses, a *p*-value < 0.10 was included in the next multivariate analysis, whereas a *p*-value < 0.05 was considered statistically significant in the remaining data. Statistical analyses were performed with IBM SPSS Statistics (version 26.0) and R software (version 4.1.2). We used several tools within the R environment, including “sampling,” “rms,” “foreign,” “rmda,” “ggplot2,” “survival,” “plyr,” “MASS,” “leaps,” “glmnet,” “pec,” “riskRegression,” and “regplot.”

## Results

### Patient characteristics

A total of 476 patients were enrolled in the study and captured with images of both NCCT and CTP. Table 1 shows the clinical information of the overall patients. The median age of the overall patients was 62 years, with women accounting for 26.5% of the total number of patients. The proportion of patients with admission BI less than or equal to 60 was 42.4%. After treatment with endovascular thrombolysis or mechanical thrombectomy, the proportion of patients with discharge BI less than or equal to 60 was decreased to 21.2%, demonstrating the effectiveness of the intervention. The median time for hospitalization was 9 days. Other clinical materials, including dyslipidemia, cerebral infarction, atrial fibrillation, hyper-homocysteinemia, diabetes, hypertension, brain injury, smoking, and complication, were also shown in the statistical table. All samples were divided into the training set (*n* = 381) and the testing set (*n* = 95), in which the number of people with good outcomes was 307 and 68, respectively. No significant difference was found between the training set and the testing set for all characteristics.

**TABLE 1:** Baseline characteristics in study sample (*n* = 476).

Clinical characteristics	Overall (*n* = 476)	Training set (*n* = 381)	Testing set (*n* = 95)	*P*
Age (year)	62 (55, 68)	62 (55, 68)	62 (56, 68)	0.719
Female sex	126 (26.5%)	107 (22.5%)	19 (4.0%)	0.317
Admission BI rating				0.696
Bad [1; 2]	202 (42.4%)	170 (35.7%)	32 (6.7%)	
Good [3; 4]	274 (57.5%)	211 (44.3%)	63 (13.2%)	
Dyslipidemia	120 (25.2%)	100 (21.0%)	20 (4.2%)	0.110
Stroke history	118 (24.8%)	95 (20.0%)	23 (4.8%)	0.515
Atrial fibrillation	18 (3.8%)	15 (3.2%)	3 (0.6%)	0.956
Hyper-homocysteinemia	94 (19.7%)	80 (16.8%)	14 (2.9%)	0.426
Diabetes	145 (30.5%)	109 (22.9%)	36 (7.6%)	0.312
Hypertension	281 (59.0%)	221 (46.4%)	60 (12.6%)	0.251
Brain injury	2 (0.4%)	2 (0.4%)	0 (0.0%)	1.000
Smoking	212 (44.5%)	173 (36.3%)	39 (8.2%)	0.395
Complications	125 (26.3%)	105 (22.1%)	20 (4.2%)	0.593
Endovascular thrombolysis	92 (19.3%)	74 (15.5%)	18 (3.8%)	0.853
Mechanical thrombectomy	58 (12.2%)	41 (8.6%)	17 (3.6%)	0.427
Conservative treatment	326 (68.5%)	266 (55.9%)	60 (12.6%)	0.525
Discharge BI rating				0.965
Bad [1; 2]	101 (21.2%)	77 (16.2%)	24 (5.0%)	
Good [3; 4]	375 (78.8%)	307 (64.5%)	68 (14.3%)	
Time for hospitalization (day)	9 (6, 11)	8 (6, 11)	9 (7, 11)	0.296

### Predicting discharge Barthel index rating of patients

#### Clinical outcome prediction (discharge BI > 60 vs. BI ≤ 60)

A total of six variables (BI_admission_score, Overall_ASPECTS, R_M2_HU, L_L_HU, Tmax_8, and cbf_38) were selected to establish the nomogram prediction model, and the model shows a strong predictive ability under the evaluation of the ROC curves, calibration curves, decision curves, and clinical impact curves.

Univariable logistic analysis and multivariable logistic analysis were made in turn by combining statistically different variables into the regression equation and stepwise modality was chosen to select the optimal variables associated with the discharge BI. The initial regression model contained eight variables (Supplementary Tables 1, 2), meeting the requirement of the Hosmer–Lemeshow goodness-of-fit test (*p* = 0.206 > 0.05). However, some continuous variables showed strong collinearity (Supplementary Figure 1), which may limit the predicted performance of the model. When removing two covariant variables, the final regression model was constructed, satisfying the Hosmer–Lemeshow goodness-of-fit test (*p* = 0.078 > 0.05).

As seen in Table 2, BI_admission_score (BI of admission), Overall_ASPECTS (minimum ASPECTS of left and right brain), R_M2_HU (mean CT intensity of the right extrainsular cortex), L_L_HU (mean CT intensity of the left lenticular nucleus), Tmax_8 (Tmax with a threshold of >8 s), and cbf_38 (CBF with a threshold of <38%) were finally selected to establish the regression model, in which the crude odds ratio (OR) indicated the univariable analysis result and the adjusted OR indicated the multivariable analysis result. The adjusted OR also showed by a forest map (Supplementary Figure 2) demonstrating that Tmax_8 limited good outcomes, whereas the others promoted good outcomes, which was consistent commonly. The higher the Overall_ASPECTS and BI_admission score, the lower the severity of the infarction.

**TABLE 2:** Multivariable logistic analysis of clinical and imaging variables with clinical outcome (BI > 60 vs. BI ≤ 60).

Characteristics	Crude OR (95% CI)	Adjusted OR (95% CI)	*P* (Wald’s test)	*P* (LR-test)
BI_admission _score	1.08 (1.06–1.10)	1.07 (1.05–1.09)	<0.001	<0.001
Overall _ASPECTS	1.59 (1.39–1.81)	1.26 (1.03–1.54)	0.027	0.027
R_M2_HU	1.18 (1.11–1.25)	1.01 (0.92–1.10)	0.862	0.862
L_L_HU	1.36 (1.24–1.48)	1.07 (0.94–1.21)	0.297	0.285
Tmax_8	0.99 (0.99–1.00)	0.99 (0.99–1.00)	0.019	0.025
cbf_38	0.98 (0.97–0.99)	1.01 (0.99–1.02)	0.378	0.402

**FIGURE 2:**
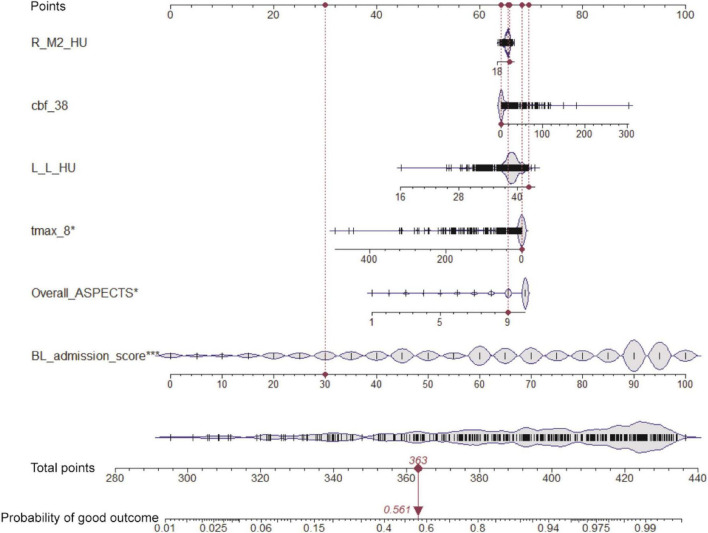
Nomogram for predicting the clinical outcome. Six variables are included in the nomogram model. For the given sample shown above in the red line, each variable has a point, and the total point reflected the probability of a good outcome (discharge BI > 60).

**FIGURE 3:**
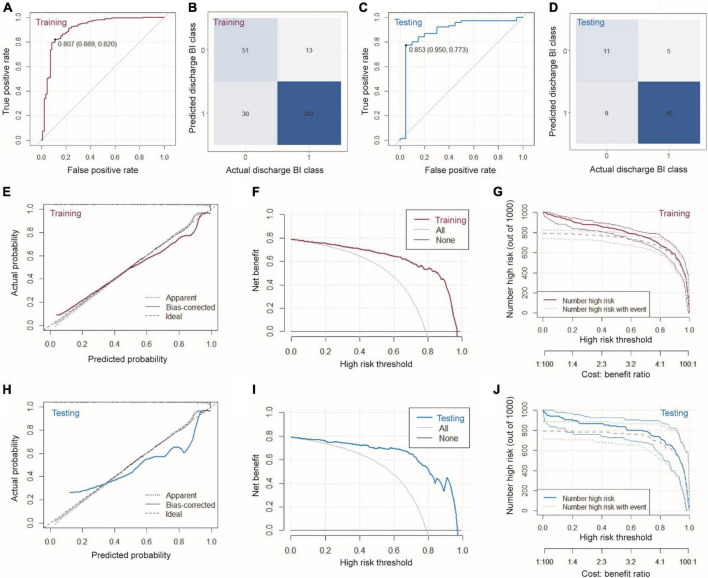
Characterizations of the nomogram model. **(A)** ROC, **(B)** confusion matrix, **(E)** calibration curve, **(F)** decision curve, and **(G)** clinical impact curve of the model on the training dataset. **(C)** ROC, **(D)** confusion matrix, **(H)** calibration curve, **(I)** decision curve, and **(J)** clinical impact curve of the model on the testing dataset.

Based on the six variables selected above, we constructed a nomogram to predict clinical outcomes (Figure 2). For a given patient, every variable corresponded to a point, and the total point corresponded to the probability of a good outcome (discharge BI > 60). To evaluate the performance of the nomogram model, a range of parameters were performed in both training and testing sets, including qualitative visualization and quantitative analysis. For qualitative display, ROC curves, calibration curves, decision curves, and clinical impact curves of the training and testing sets are illustrated in Figure 3. Meanwhile, confusion matrixes are also shown here. Moreover, quantitative results that were performed and summarized are shown in Table 3. As shown in Figures 3A–D and Table 3, AUC values are 0.908 [95% confidence interval (CI): 0.865–0.952] and 0.883 (95% CI: 0.781–0.985) in the training and testing sets, respectively. The confusion matrixes showed the accuracy of 0.887 and 0.853 in the training and testing sets, respectively. F1-scores, combining the precision and recall of the model, were higher than 0.9 both in the training and testing sets. Figures 3E–G shows the calibration curve, the decision curve, and the clinical impact curve for the model on the training dataset. Figures 3H–J shows the corresponding curves of the model on the testing dataset. Calibration curves were used to check the quantile relationship between predictions and the actual values, and it could be easily observed that the predicted probability was similar to the actual probability. Moreover, the discriminative property was quantified by the following parameters, including D index, S/p, Brier, E_max_, and E_avg_ (Table 3). D indexes were 0.467 and 0.360 in the training and testing sets, demonstrating outstanding and good discrimination of the model. Other parameters also confirmed that the model owned good discriminative ability. Decision curves were estimates of the standardized net benefit by the probability threshold used to categorize observations as “high risk.” Both decision curves and clinical impact curves could be used to measure the clinical utility of the nomogram model.

**TABLE 3:** Quantitative analysis of nomogram model both in the training and testing sets.

	AUC [95% CI]	Accuracy	F1-score	Kappa	D	S/p	Brier	E_max_	E_avg_
Training set	0.908 [0.865, 0.952]	0.887	0.930	0.635	0.467	0.636	0.081	0.059	0.020
Testing set	0.883 [0.781, 0.985]	0.853	0.909	0.522	0.360	0.570	0.098	0.133	0.036

At a wide range of the high-risk threshold, the nomogram model owned higher clinical net benefit, both in the training and testing sets. All these results verified that the nomogram model demonstrated superior predictive performance, which would achieve high clinical benefit and help us make better clinical decisions.

#### Clinical outcome prediction (discharge Barthel index in four classes)

A model with nine features (BI_admission_score, L_L_HU, L_M1, R_M5, CBF_20, L_M4, Tmax_10, L_M3, and CBF_30) showed excellent classification accuracy under the tests of ROC curves.

**FIGURE 4:**
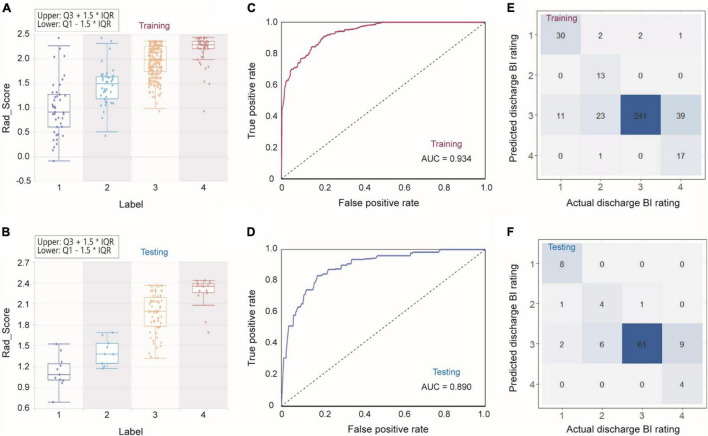
Combining Rad_Score and Bagging decision tree for predicting discharge BI with four classes. **(A,B)** Rad_Score distribution within four classes, ROC curves **(C,D)**, and confusion matrixes **(E,F)** in the training and testing sets.

To predict discharge BI further accurately, we divided discharge BI into four classes, in which 1 represented a BI score of 0–40, 2 represented a score of 41–60, 3 represented a score of 61–99, and 4 represented a score of 100. First, we used two operators to select features sequentially, i.e., *F*-test’s *p*-value of 0.05 and LASSO with α of 0.05. With the sequential execution of these two algorithms, nine features obtained are shown in Supplementary Figure 3. Considering these nine features and the corresponding weight, we could obtain an equation to compute the Rad_Score of each sample. As shown in Figures 4A,B, both in the training and testing sets, the average Rad_Score of four groups are quite different, indicating that the following classifier can achieve a good classification. It should be noted that, after classifying by using the Bagging decision tree, the AUCs were 0.934 and 0.890 for the training and testing sets, respectively (Figures 4C,D). From the confusion matrixes shown in Figures 4E,F, the accuracy can reach 0.792 and 0.802 in the training and testing sets. At the same time, kappa indexes are 0.614 and 0.661 for the training and testing sets. Importantly, we reported ROC curves for predicting each discharge BI class (Supplementary Figure 4), and the AUCs are also listed in Supplementary Table 3. It should be noted that AUCs for classifying every discharge BI class were higher than 0.850, confirming that nine features combined with the Bagging decision tree classifier could achieve excellent classification performance.

### Estimating the time for hospitalization of patients

After comparing the COX, BSR, and LASSO models, the LASSO model was adopted for fewer data and a higher consistency index. On this basis, the nomogram model we established can accurately predict the length of hospital stay.

To predict the time for patients to achieve a good outcome, that is, the time for hospitalization, we constructed and compared three models to get the best predictive performance. Based on the optimal model, we reported the hazard ratios (HRs) and constructed a nomogram to predict the time for hospitalization.

The first one was the COX model, in which the feature selection was using univariate and multivariable Cox regression analyses orderly. From Supplementary Table 4, a number of variables showed statistical differences in univariate Cox regression. Then, a multivariable Cox regression analysis was performed; finally, 12 features (BI_admission_score, atrial_fibrillation, therapy, Left_ASPECTS, L_M2, R_M1_HU, L_M1_HU, Tmax_10, Tmax_8, Tmax_6, cbv_42, and cbv_38) were selected for constructing the COX model. The second one was the BSR model. On the basis of the maximum adjusted *R*^2^ and the minimum AIC, 3 features were finally selected (BI_admission_score, complication, and Overall_ASPECTS) and used to establish the BSR model. The third one was the LASSO model, in which BI_admission_score, Overall_ASPECTS, and Tmax_10 were selected. The features selected from the three models underwent the Cox regression analysis.

**FIGURE 5:**
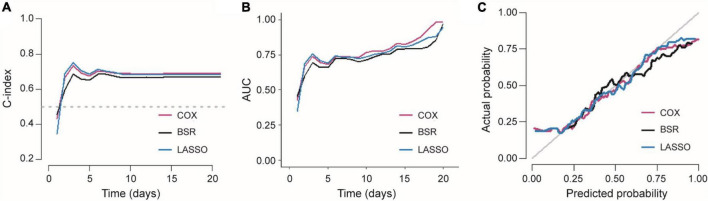
Comparison of three Cox proportional hazard models. **(A)** C-index and **(B)** AUC of three models varied with time. **(C)** Calibration curves of three Cox proportional hazard models.

When comparing the performance of three models, the C-index, AUC, and calibration curve are plotted in Figure 5. All these results demonstrated that LASSO was the best model due to its higher C-index and fewer features. As mentioned above, the number of features in COX, BSR, and LASSO was 12, 3, and 3, respectively. Meanwhile, the C-index of these three models were 0.704 (SE = 0.018), 0.682 (SE = 0.018), and 0.700 (SE = 0.019). It is important to note that there was no significant difference between LASSO and COX. Thus, we chose the LASSO model (BI_admission_score, Overall_ASPECTS, and Tmax_10) to predict the time to achieve a good outcome. Based on LASSO features, the detailed information of the final Cox proportional hazard model is listed in Table 4.

**TABLE 4:** Cox proportional hazard model based on the features selected by LASSO.

Characteristics	Crude HR (95% CI)	Adjusted HR (95% CI)	*z*	*P*
BI_admission_score	1.02 (1.02–1.03)	1.02 (1.02–1.03)	7.87	<0.001
Overall_ASPECTS	1.27 (1.16–1.38)	1.18 (1.07–1.29)	3.31	<0.001
Tmax_10	0.99 (0.99–1.00)	0.999 (0.996–1.002)	−0.68	0.494

**FIGURE 6:**
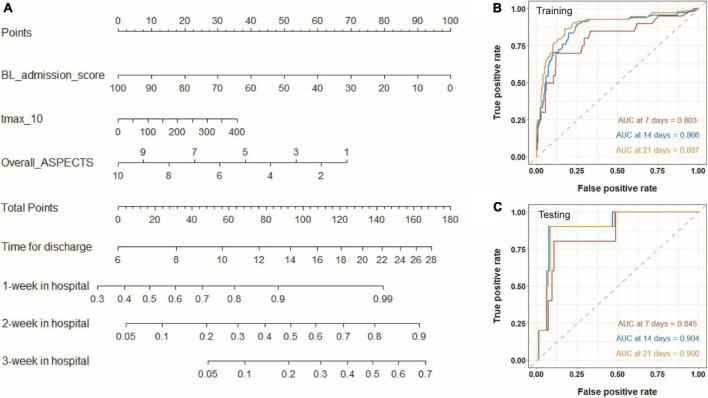
Nomogram for predicting the time for achieving a good outcome. **(A)** Three variables from Cox proportional hazard regression model are included into the nomogram model. For a given sample, each variable has a point, and the total point reflected the time for discharge and the probability of the patient staying 1, 2, or 3 weeks in hospital. Panels **(B,C)** show time-dependent ROC curves of the model for the training and testing sets.

On the basis of the selected Cox proportional hazard model, we established a nomogram to predict the time for hospitalization of patients and the probability of the patient staying 1, 2, or 3 weeks in hospital (Figure 6A). With this nomogram, we could easily obtain the time to realize a good outcome (BI > 60). As shown in Figures 6B,C, AUC for predicting discharge at specific times (7, 14, and 21 days) are higher than 0.80, both in the training and testing sets, demonstrating that the nomogram owned an excellent ability to predict the length of hospital stay. Calibration curves are also plotted several times and shown in Supplementary Figure 5. High AUC values and good calibration capability demonstrated that the final Cox proportional hazard model held great potential in predicting the time for achieving a good outcome.

## Discussion

In our study, predictive nomograms for the short-term prognosis in patients with ischemic stroke was established by NCCT- and CTP-based imaging features and clinical features. The established nomograms were tested with both training set and testing set, and the results showed that the models were able to accurately classify a patient’s BI (the AUC criteria for discharge suggestion were defined as 0.850) and predict the time duration of hospital stay (discharge at a specific time [7, 14, or 21 days] on the basis of defined AUC criteria [0.800]). Thus, we believe that the proposed method can predict the short-term prognosis of patients with stroke accurately, thereby assisting physicians to optimize personalized medical management strategies.

A total of 476 patients were included in this study. To our knowledge, the amount of data included in this experiment is the largest in the AI-assisted diagnosis of stroke experiments in the past 10 years (Bentley et al., 2014; Lucas et al., 2018; Ho et al., 2019; Kasasbeh et al., 2019; Xie et al., 2019; Bacchi et al., 2020), which provided an adequate quantitative base for model development, thus making the models more stable and objective. The male-to-female ratio included in this study was about 1:2.8, which is consistent with the incidence of stroke in Northeast China, where the incidence of stroke in men is significantly higher than in women. At this stage, there are various measurement tools that could be employed to evaluate the ADL of patients with stroke. Although the BI scale showed no difference or improvement in results as compared with other scales, the BI scale did present the advantages of high sensitivity, comprehensive content, optimal operability, clear scoring, and good reliability and validity in the application conditions of both face-to-face interview and telephone visits. Other risk factors related to stroke, such as diabetes, smoking status, atrial fibrillation, and infarction history, were also evaluated in our study (Pistoia et al., 2016; Boehme et al., 2017; Zhang et al., 2020), which was crucial for the subsequent extraction of clinical features, the combination of clinical features and imaging features, and the objective expression of the nomogram.

According to the abovementioned experimental procedures, we developed a nomogram based on independent clinical factors and imaging characteristics of NCCT and CTP in patients with ischemic stroke, which has been externally validated as a predictive tool for visualized pretreatment prognosis prediction. Compared with other studies, that paid more attention to the evolution of stroke lesions, we focused on the prediction regarding the behavioral abilities of patients, thereby indicating functional outcomes in stroke trials. For example, Pinto et al. (2018) predicted stroke lesion outcomes of 75 patients based on MRI combined with clinical information. Ho et al. (2019) made predictions on the possible physiological changes in ischemic stroke tissues using a deep convolutional neural network (CNN) on source magnetic resonance perfusion images. Lucas et al. (2018) employed a dataset of 29 subjects to predict ischemic stroke evolution based on acute CTP [CBV and time to peak (TTP)] data by interpolating low-dimensional shape presentations. Xie et al. (2019) built extreme gradient boosting (XGB) and gradient boosting machine (GBM) models to predict the modified Rankin scale (mRS) scores at 90 days on the basis of NCCT and CTP images [mean transit time (MTT) and CBV] for binary prediction of an mRS score, in which XGB and GBM models presented AUC of 0.746 and 0.748, respectively. Tang et al. (2020) developed a radiomic signature using perfusion-weighted imaging (CBF and Tmax) as prognostic biomarkers to validate the radiomic nomogram for predicting clinical outcomes of thrombolysis in patients with acute ischemic stroke. On the contrary, our nomogram model focused on the more optimal performance in the binary classification of the BI scale. In addition, a variety of parameters were collected from CTP maps, including CBV, CBF, Tmax, and mismatch, and mismatch ratio so as to extract more effective parameters, which reflected the advantages of CTP through the mismatch area. As compared with previous studies on radiomics and machine learning conducted in stroke settings (Bentley et al., 2014; Ho et al., 2017, 2019; Peter et al., 2017; Lucas et al., 2018; Kasasbeh et al., 2019; Qiu et al., 2019; Xie et al., 2019; Bacchi et al., 2020; Robben et al., 2020), we conducted a more comprehensive evaluation to verify the developed predictive tool by assessing the discrimination ability, calibration, and clinical utility, after which a more refined nomogram model was obtained. In addition, compared with previous experiments that only took lesions as the research object, we focused on the entire brain, that is, the extracted imaging features cover both the left and right brains. Even if the lesion is located in the left brain, some characteristics of the right brain will have a certain impact on the clinical prognosis of the patient. This is also one of the strengths of our experiment.

As far as we know, the prediction of the time duration of hospitalization in patients with stroke is still a relatively new research point. The practical application value can be attributed to three points. First, prediction of the reasonable length of hospitalization may assist the physicians to determine the hospital stay of individuals by indicating that extended hospital stay may have less benefit on the patient based on the BI analyses, thereby improving the efficiency of allocation of medical resources, especially in developing countries where limited clinical resources are available. Second, this may be helpful in prompting the patient to start rehabilitation treatment, and in the case of remission, the rehabilitation can be carried out as soon as possible, which is helpful for the recovery of the patient’s daily behavior. Third, the predicted hospital stay can provide rough estimates for disease severity and hospitalization expenses, which may be referred by the patient before hospitalization. In clinical practice, it is quite common that certain families may be not able to afford endovascular intervention and would prefer conservative treatment due to economic considerations. According to our analyses, no significant difference was discovered between patients with ischemic stroke receiving the endovascular intervention (thrombectomy, stenting, or balloon dilation) and those receiving medicinal thrombolysis regarding the duration of time for hospitalization, which may suggest that the choice of treatment method for ischemia stroke had no significant impact on short-term BI, while further studies are still indicated to explore the impacts of selected treatments on long-term prognosis and recovery of brain function.

Despite the optimal performance of the nomogram models mentioned above, our study has limitations. First of all, since the study design was a retrospective analysis, potential selection bias was inevitable. Second, all the enrolled patients came from a single stroke center. Nevertheless, data from two scanners were utilized, which guaranteed the stability of the model to a certain extent. Third, features were extracted based on specific platforms and specific thresholds, which may have an impact on the generalization of our models. However, the nomogram showed promising performance, indicating its potential application in a broader practice. Thus, further multicenter trials with fine designs are warranted to verify the encouraging results obtained from our study, thereby promoting the model application in a diverse clinical practice.

## Conclusion

In conclusion, our study suggested that signatures may have the potential to predict the short-term prognosis of patients with ischemic stroke. The results proved that nomograms based on features from NCCT, and CTP images demonstrated an exciting ability to make individualized predictions for clinical outcomes in an ischemic stroke setting. Different from traditional imaging evaluation and patient physical assessment, the methodology provides a new direction to improve clinical diagnosis and treatment efficiency in the future.

Nomogram models demonstrated superior personalized clinical outcome predictive performance in patients’ ADL and the length of hospital stay, which shows great potential in improving the clinical efficiency and may assist the physicians to optimize the long-term rehabilitation management programs based on the expected recovery of the patients.

## Data availability statement

The original contributions presented in this study are included in the article/Supplementary Material, further inquiries can be directed to the corresponding author.

## Ethics statement

The studies involving human participants were reviewed and approved by the Ethics Committee of China-Japan Union Hospital of Jilin University. Written informed consent for participation was not required for this study in accordance with the national legislation and the institutional requirements.

## Author contributions

HS: drafting the manuscript. JW: devising the manuscript critically for important intellectual content and design of methodology. QZ: analysis and interpretation of data. LL and ZL: provision of study materials. XZ: conducting a research and investigation process. HY and YS: scrub data and maintain research data. SL and FS: oversight and leadership responsibility for the research activity planning and execution. ZM: formulation of overarching research goals and aims. All authors contributed to the article and approved the submitted version.

## Conflict of interest

JW, QZ, HY, YS, SL, and FS were employed by Shanghai United Imaging Intelligence Co., Ltd. The remaining authors declare that the research was conducted in the absence of any commercial or financial relationships that could be construed as a potential conflict of interest. The reviewer, JS, declared a past co-authorship with several of the authors, SL and FS to the handling editor.

## Publisher’s note

All claims expressed in this article are solely those of the authors and do not necessarily represent those of their affiliated organizations, or those of the publisher, the editors and the reviewers. Any product that may be evaluated in this article, or claim that may be made by its manufacturer, is not guaranteed or endorsed by the publisher.

## Footnotes

1
https://www.uii-ai.com/en/uai/scientific-research

## Supplementary material

Supplementary Figure 1 Correlation analysis of continuous variables in the initial regression model. **(A)** The correlation coefficient between any two variables from R_M2_HU, L_C_HU, L_L_HU, and L_M2_HU. Both scatter diagrams and histograms represent the distribution of the variables. **(B)** The heat map qualitatively shows the correlation, in which the correlation strength was positively related to the intensity and the size.

Supplementary Figure 2 Forest map showing the adjusted OR of variables in the multivariable regression model.

Supplementary Figure 3 Features selection by LASSO. **(A)** The changes of AUC and features’ number with −log(Alpha). **(B)** Coefficients of nine features vary with −log(Alpha). **(C)** Coefficients of nine features at a designated α = 0.05.

Supplementary Figure 4 Receiver operating characteristics (ROC) curves for predicting discharge BI rating into four classes both in the training set **(A)** and the testing set **(B)**.

Supplementary Figure 5 Calibration curves for predicting the time to get a good outcome (BI > 60).

Supplementary Equation 1 Rad_Score equation composed of nine features and corresponding coefficients was used to classify the discharge BI into four classes.

Rad_Score= 0.3858*BI_admission_score+ 0.0074*L_L_HU- 0.0061*L_M1- 0.0092*R_M5- 0.0210C*BF_20"-0.0314*L_M4-0.0509*Tmax_10-0.0541*L_M3-0.0684*CBF_30+ 1.8403"

## Abbreviations

NCCT: non-contrast CT
CTP: CT perfusion
ADL: activity of daily living
GBD: Global Burden of Diseases
BI: Barthel index
ASPECTS: Alberta stroke program early CT score
CBF: cerebral blood flow
CBV: cerebral blood volume
MTT: mean transit time
TTP: time to peak
LASSO: least absolute shrinkage and selection operator
BSR: best subset regression
AIC: Akaike information criterion
CNN: convolutional neural network
ANN: artificial neural networks
XGB: extreme gradient boosting
GBM: gradient boosting machine
ROC: receiver operating characteristics
AUC: area under the curve
C-index: concordance index
OR: odds ratio
HR: hazard ratio
CI: confidence interval.
