# Supplementary material for: Nomograms predict prognosis and hospitalization time using non-contrast CT and CT perfusion in patients with ischemic stroke
Source: Front Neurosci. 2022 Jul 22;16:912287. doi: 10.3389/fnins.2022.912287 (PMC9355636; doi:10.3389/fnins.2022.912287)
Supplement: Supplementary Figure 1 — Correlation analysis of continuous variables in the initial regression model. (A) The correlation coefficient between any two variables from R_M2_HU, L_C_HU, L_L_HU, and L_M2_HU. Both scatter diagrams and histograms represent the distribution of the variables. (B) The heat map qualitatively shows the correlation, in which the correlation strength was positively related to the intensity and the size. [file Data_Sheet_1.docx]

**SUPPLEMENTARY MATERIALS**

**Supplementary Table 1. Univariate associations of clinical and imaging variables with clinical outcome (BI > 60 *vs.* BI ≤ 60).**

| **Number** | **Characteristics** | **OR** | **95% CI** | ***P*** |
| --- | --- | --- | --- | --- |
| 1 | BI_admission_score | 1.08 | 1.06 - 1.10 | < 0.001 |
| 2 | atrial_fibrillation | 0.16 | 0.06 - 0.47 | 0.001 |
| 3 | complication | 0.07 | 0.04 - 0.12 | < 0.001 |
| 4 | therapy | 0.31 | 0.18 - 0.54 | < 0.001 |
| 5 | Left_ASPECTS | 1.87 | 1.52 - 2.31 | < 0.001 |
| 6 | Right_ASPECTS | 1.20 | 1.04 - 1.40 | 0.016 |
| 7 | Overall_ASPECTS | 1.59 | 1.39 - 1.81 | < 0.001 |
| 8 | R_M1 | 0.39 | 0.13 - 1.12 | 0.080 |
| 9 | R_M3 | 0.19 | 0.07 - 0.53 | 0.001 |
| 10 | R_M4 | 0.42 | 0.18 - 1.00 | 0.050 |
| 11 | R_M5 | 0.48 | 0.21 - 1.12 | 0.090 |
| 12 | R_M6 | 0.43 | 0.19 - 0.97 | 0.042 |
| 13 | L_C | 0.15 | 0.04 - 0.66 | 0.012 |
| 14 | L_IC | 0.07 | 0.02 - 0.19 | < 0.001 |
| 15 | L_L | 0.28 | 0.11 - 0.70 | 0.007 |
| 16 | L_I | 0.31 | 0.15 - 0.68 | 0.003 |
| 17 | L_M1 | 0.04 | 0.01 - 0.16 | < 0.001 |
| 18 | L_M2 | 0.09 | 0.03 - 0.26 | < 0.001 |
| 19 | L_M3 | 0.05 | 0.01 - 0.19 | < 0.001 |
| 20 | L_M4 | 0.09 | 0.04 - 0.23 | < 0.001 |
| 21 | L_M5 | 0.07 | 0.03 - 0.17 | < 0.001 |
| 22 | L_M6 | 0.08 | 0.03 - 0.22 | < 0.001 |
| 23 | R_M1_HU | 1.22 | 1.14 - 1.31 | < 0.001 |
| 24 | R_M2_HU | 1.18 | 1.11 - 1.25 | < 0.001 |
| 25 | R_M3_HU | 1.29 | 1.19 - 1.40 | < 0.001 |
| 26 | L_C_HU | 1.31 | 1.21 - 1.41 | < 0.001 |
| 27 | L_IC_HU | 1.41 | 1.27 - 1.56 | < 0.001 |
| 28 | L_L_HU | 1.36 | 1.24 - 1.48 | < 0.001 |
| 29 | L_I_HU | 1.34 | 1.23 - 1.46 | < 0.001 |
| 30 | L_M1_HU | 1.21 | 1.13 - 1.29 | < 0.001 |
| 31 | L_M2_HU | 1.17 | 1.10 - 1.24 | < 0.001 |
| 32 | L_M3_HU | 1.26 | 1.16 - 1.37 | < 0.001 |
| 33 | Tmax_10 | 0.99 | 0.98 - 0.99 | < 0.001 |
| 34 | Tmax_8 | 0.99 | 0.99 - 0.99 | < 0.001 |
| 35 | Tmax_6 | 1.00 | 0.99 - 1.00 | < 0.001 |
| 36 | Tmax_4 | 1.00 | 1.00 - 1.00 | 0.001 |
| 37 | cbf_38 | 0.98 | 0.97 - 0.99 | < 0.001 |
| 38 | cbf_34 | 0.97 | 0.96 - 0.98 | < 0.001 |
| 39 | cbf_30 | 0.96 | 0.94 - 0.97 | < 0.001 |
| 40 | cbf_20 | 0.92 | 0.89 - 0.95 | < 0.001 |
| 41 | cbv_42 | 0.97 | 0.96 - 0.98 | < 0.001 |
| 42 | cbv_38 | 0.96 | 0.94 - 0.97 | < 0.001 |
| 43 | cbv_34 | 0.94 | 0.92 - 0.97 | < 0.001 |
| 44 | mismatch_volume | 1.00 | 0.99 - 1.00 | < 0.001 |

**Supplementary Table 2. Multivariable associations of clinical and imaging variables with clinical outcome (BI > 60 *vs.* BI ≤ 60).**

| **Characteristics** | **Crude OR (95% CI)** | **Adjusted OR (95% CI)** | ***P* (Wald’s test)** | ***P* (LR-test)** |
| --- | --- | --- | --- | --- |
| BI_admission_score | 1.08 (1.06 - 1.10) | 1.08 (1.06 - 1.10) | < 0.001 | < 0.001 |
| Overall_ASPECTS | 1.59 (1.39 - 1.81) | 1.19 (0.96 - 1.46) | 0.111 | 0.110 |
| R_M2_HU | 1.18 (1.11 - 1.25) | 1.13 (0.98 - 1.30) | 0.104 | 0.104 |
| L_C_HU | 1.31 (1.21 - 1.41) | 0.83 (0.63 - 1.09) | 0.171 | 0.168 |
| L_L_HU | 1.36 (1.24 - 1.48) | 1.28 (1.01 - 1.60) | 0.037 | 0.036 |
| L_M2_HU | 1.17 (1.10 - 1.24) | 0.91 (0.78 - 1.06) | 0.239 | 0.238 |
| Tmax_8 | 0.99 (0.99 - 1.00) | 0.99 (0.99 - 1.00) | 0.006 | 0.008 |
| cbf_38 | 0.98 (0.97 - 0.99) | 1.01 (0.99 - 1.02) | 0.414 | 0.434 |

**Supplementary Table 3. AUC for predicting discharge BI rating in four classes both in the training and testing sets.**

|  | **AUC (95% CI) of training set** | **AUC (95% CI) of testing set** |
| --- | --- | --- |
| Discharge BI rating = 1 | 0.954 [0.914, 0.994] | 0.975 [0.942, 1.000] |
| Discharge BI rating = 2 | 0.910 [0.860, 0.960] | 0.888 [0.815, 0.960] |
| Discharge BI rating = 3 | 0.850 [0.809, 0.892] | 0.878 [0.803, 0.952] |
| Discharge BI rating = 4 | 0.879 [0.832, 0.925] | 0.909 [0.817, 1.000] |

**Supplementary Table 4. Univariate Cox regression analysis (status = discharge BI > 60).**

| **Characteristics** | **HR** | **95% CI** | ***P* value** |
| --- | --- | --- | --- |
| BI_admission_score | 1.02 | 1.02 - 1.03 | < 0.001 |
| atrial_fibrillation | 0.31 | 0.14 - 0.71 | 0.005 |
| complications | 0.36 | 0.26 - 0.50 | < 0.001 |
| therapy | 0.50 | 0.36 - 0.70 | < 0.001 |
| Left_ASPECTS | 1.35 | 1.16 - 1.57 | < 0.001 |
| Right_ASPECTS | 1.16 | 1.06 - 1.28 | 0.002 |
| Overall_ASPECTS | 1.27 | 1.16 - 1.38 | < 0.001 |
| R_IC | 0.64 | 0.37 - 1.09 | 0.099 |
| R_I | 0.62 | 0.41 - 0.93 | 0.021 |
| R_M1 | 0.37 | 0.18 - 0.76 | 0.007 |
| R_M2 | 0.58 | 0.32 - 1.03 | 0.062 |
| R_M3 | 0.43 | 0.20 - 0.91 | 0.027 |
| R_M4 | 0.45 | 0.26 - 0.77 | 0.004 |
| R_M5 | 0.63 | 0.38 - 1.05 | 0.078 |
| L_IC | 0.31 | 0.13 - 0.75 | 0.010 |
| L_L | 0.51 | 0.27 - 0.96 | 0.038 |
| L_M1 | 0.26 | 0.06 - 1.03 | 0.056 |
| L_M2 | 0.37 | 0.15 - 0.90 | 0.029 |
| L_M3 | 0.19 | 0.06 - 0.58 | 0.004 |
| L_M4 | 0.32 | 0.15 - 0.68 | 0.003 |
| L_M5 | 0.31 | 0.14 - 0.65 | 0.002 |
| L_M6 | 0.26 | 0.12 - 0.58 | 0.001 |
| R_M1_HU | 1.06 | 1.03 - 1.10 | 0.001 |
| R_M2_HU | 1.04 | 1.01 - 1.07 | 0.018 |
| R_M3_HU | 1.07 | 1.03 - 1.12 | < 0.001 |
| L_C_HU | 1.07 | 1.03 - 1.11 | 0.002 |
| L_IC_HU | 1.09 | 1.03 - 1.14 | 0.001 |
| L_L_HU | 1.08 | 1.03 - 1.13 | 0.001 |
| L_I_HU | 1.07 | 1.03 - 1.12 | 0.002 |
| L_M3_HU | 1.05 | 1.01 - 1.09 | 0.013 |
| Tmax_10 | 0.99 | 0.99 - 1.00 | 0.001 |
| Tmax_8 | 1.00 | 0.99 - 1.00 | 0.001 |
| Tmax_6 | 1.00 | 1.00 - 1.00 | 0.001 |
| Tmax_4 | 1.00 | 1.00 - 1.00 | 0.007 |
| cbf_38 | 0.99 | 0.98 - 1.00 | 0.002 |
| cbf_34 | 0.99 | 0.98 - 0.99 | 0.001 |
| cbf_30 | 0.98 | 0.97 - 0.99 | < 0.001 |
| cbf_20 | 0.95 | 0.93 - 0.98 | < 0.001 |
| cbv_42 | 0.99 | 0.98 - 0.99 | 0.002 |
| cbv_38 | 0.98 | 0.96 - 0.99 | < 0.001 |
| cbv_34 | 0.97 | 0.95 - 0.99 | < 0.001 |
| mismatch_volume | 1.00 | 1.00 - 1.00 | 0.003 |


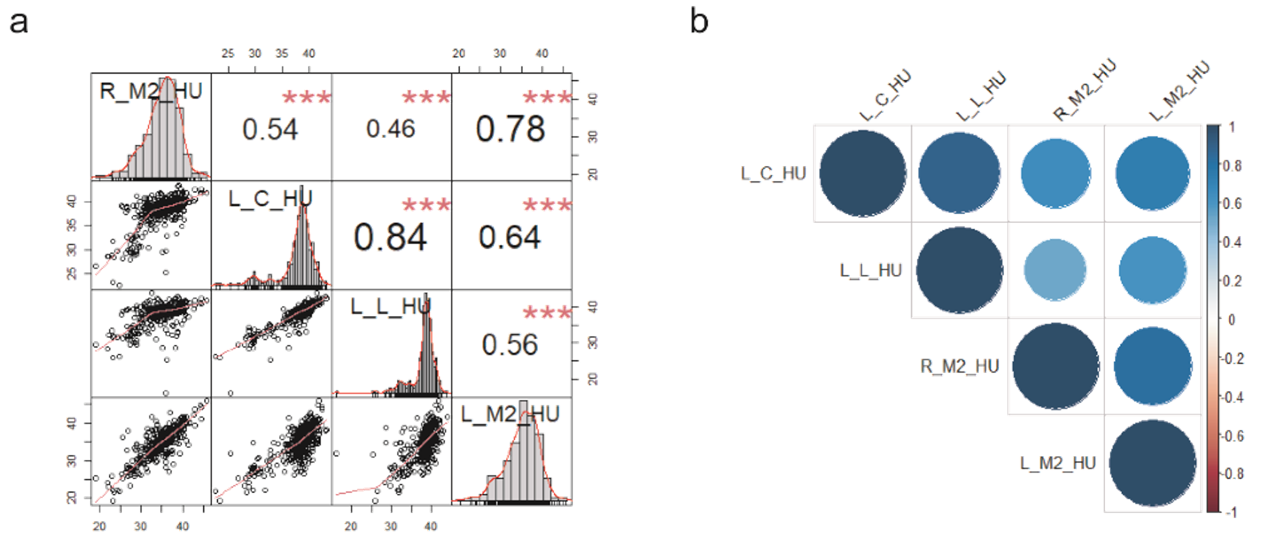
**Supplementary Figure 1. Correlation analysis of continuous variables in the initial regression model.** (a) The correlation coefficient between any two variables from R_M2_HU, L_C_HU, L_L_HU and L_M2_HU. Both scatter diagrams and histogram represented the distribution of the variables. (b) The heat map qualitatively showing the correlation, in which the correlation strength was positively related to the intensity and the size.


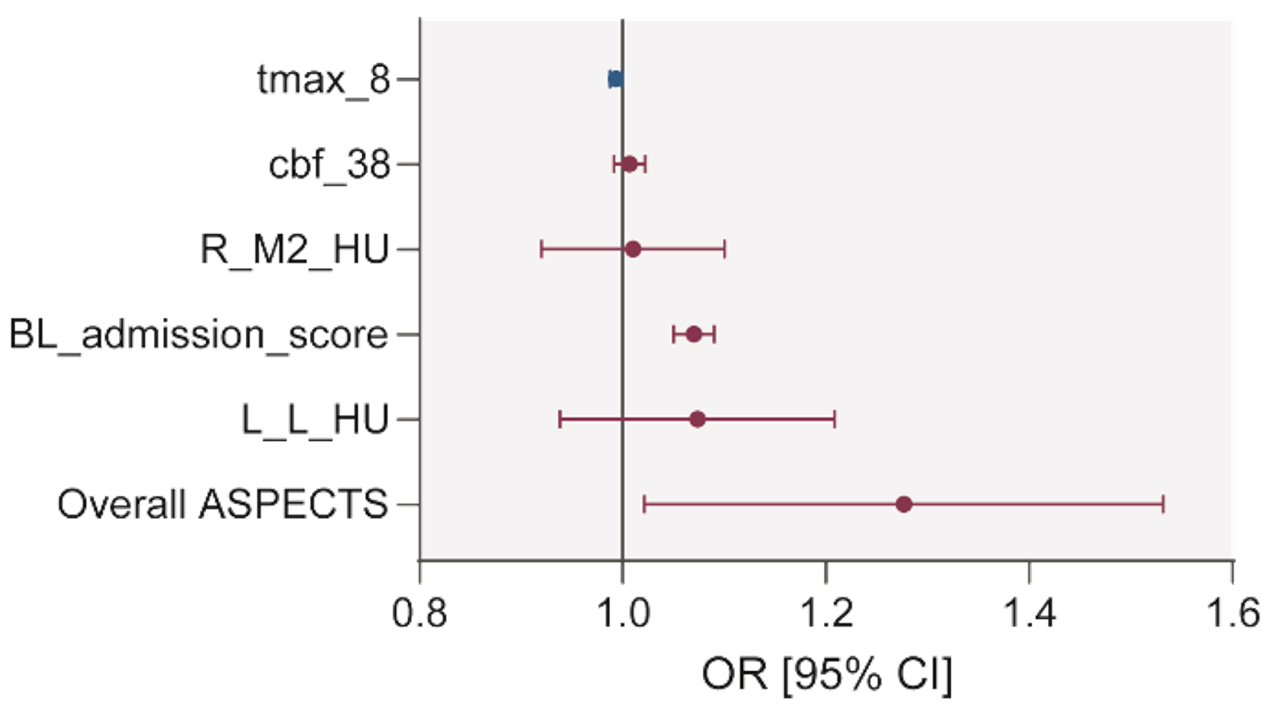
**Supplementary Figure 2. Forest map showing the adjusted OR of variables in the multivariable regression model.**


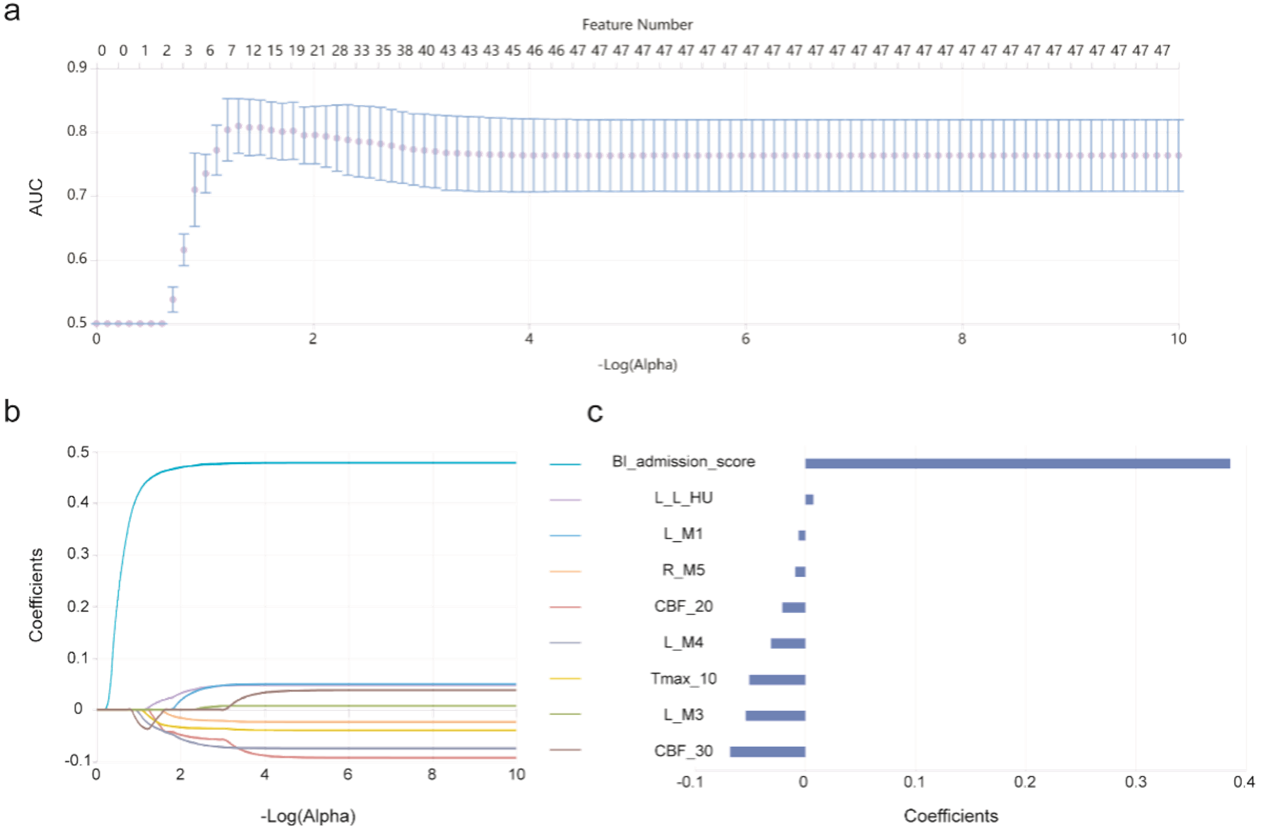
**Supplementary Figure 3. Features selection by LASSO.** (a) The changes of AUC and features’ number with -log(Alpha). (b) Coefficients of nine features varying with -log(Alpha). (c) Coefficients of nine features at a designated α = 0.05.


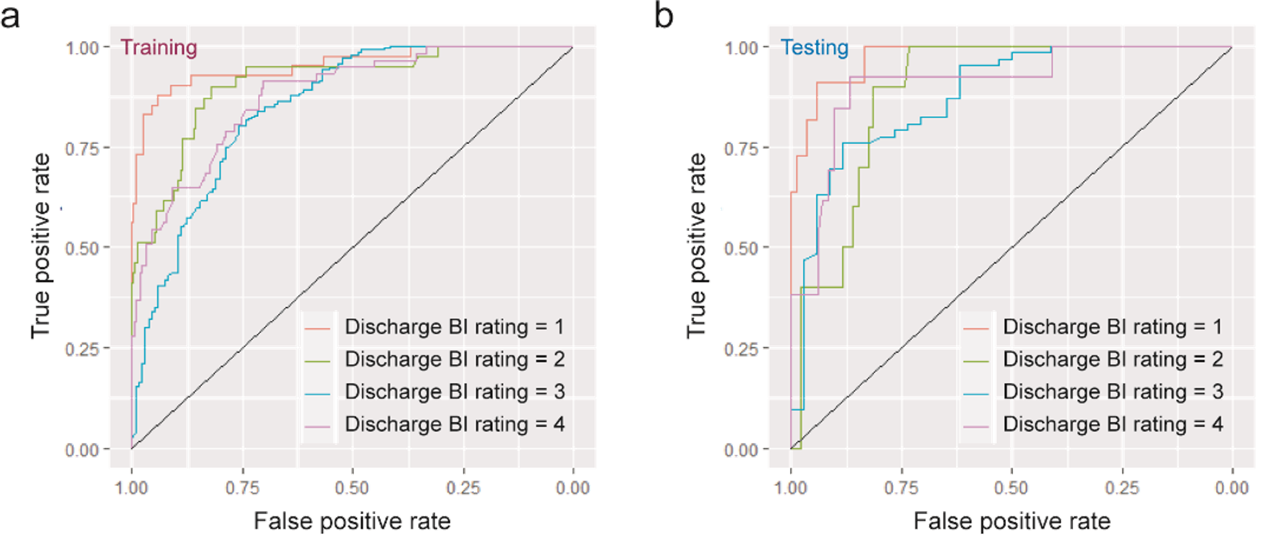
**Supplementary Figure 4. ROC curves for predicting discharge BI rating into four classes both in training set (a) and testing set (b).**


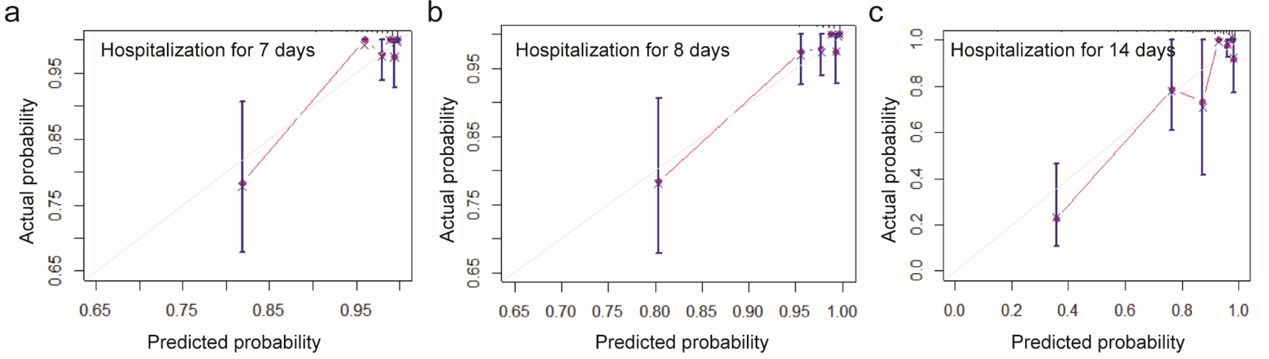
**Supplementary Figure 5. Calibration curves for predicting the time to get good outcome (BI > 60).**
